# Low pH immobilizes and kills human leukocytes and prevents transmission of cell-associated HIV in a mouse model

**DOI:** 10.1186/1471-2334-5-79

**Published:** 2005-09-30

**Authors:** Stuart S Olmsted, Kristen V Khanna, Erina M Ng, Steven T Whitten, Owen N Johnson, Richard B Markham, Richard A Cone, Thomas R Moench

**Affiliations:** 1 Department of Biophysics, Johns Hopkins University, Jenkins Hall, 3400 N. Charles St., Baltimore, MD 21218, USA; 2 RAND Corporation, 201 N. Craig St #202, Pittsburgh, PA 15213, USA; 3 ReProtect, Inc., 703 Stags Head Rd, Baltimore, MD 21286, USA; 4 Department of Molecular Microbiology and Immunology, Johns Hopkins Bloomberg School of Public Health, 615 North Wolfe Street, Suite E5132, Baltimore, MD 21205 USA

## Abstract

**Background:**

Both cell-associated and cell-free HIV virions are present in semen and cervical secretions of HIV-infected individuals. Thus, topical microbicides may need to inactivate both cell-associated and cell-free HIV to prevent sexual transmission of HIV/AIDS. To determine if the mild acidity of the healthy vagina and acid buffering microbicides would prevent transmission by HIV-infected leukocytes, we measured the effect of pH on leukocyte motility, viability and intracellular pH and tested the ability of an acidic buffering microbicide (BufferGel^®^) to prevent the transmission of cell-associated HIV in a HuPBL-SCID mouse model.

**Methods:**

Human lymphocyte, monocyte, and macrophage motilities were measured as a function of time and pH using various acidifying agents. Lymphocyte and macrophage motilities were measured using video microscopy. Monocyte motility was measured using video microscopy and chemotactic chambers. Peripheral blood mononuclear cell (PBMC) viability and intracellular pH were determined as a function of time and pH using fluorescent dyes. HuPBL-SCID mice were pretreated with BufferGel, saline, or a control gel and challenged with HIV-1-infected human PBMCs.

**Results:**

Progressive motility was completely abolished in all cell types between pH 5.5 and 6.0. Concomitantly, at and below pH 5.5, the intracellular pH of PBMCs dropped precipitously to match the extracellular medium and did not recover. After acidification with hydrochloric acid to pH 4.5 for 60 min, although completely immotile, 58% of PBMCs excluded ethidium homodimer-1 (dead-cell dye). In contrast, when acidified to this pH with BufferGel, a microbicide designed to maintain vaginal acidity in the presence of semen, only 4% excluded dye at 10 min and none excluded dye after 30 min. BufferGel significantly reduced transmission of HIV-1 in HuPBL-SCID mice (1 of 12 infected) compared to saline (12 of 12 infected) and a control gel (5 of 7 infected).

**Conclusion:**

These results suggest that physiologic or microbicide-induced acid immobilization and killing of infected white blood cells may be effective in preventing sexual transmission of cell-associated HIV.

## Background

Most HIV transmission occurs sexually [1,2], and semen [3-6] and cervicovaginal secretions [7] contain free HIV virions as well as HIV-infected leukocytes. Whether cell-free virus or infected cells are the primary means of transmission, or whether both are important remains unknown.

Free virus transmits infection in monkey [8], chimpanzee [9], and cat [10] vaginal-challenge models, although the amount of virus used [8] has generally been greater than the amount of infectious virus found in semen of HIV-infected men [4]. Studies of cell-vectored transmission include an unsuccessful attempt to establish a model of vaginal transmission with cryopreserved SIV-infected cells in the monkey [8], but, fresh HIV-infected cells transmitted infection in the chimpanzee when applied to the cervical os [9], fresh FIV-infected cells transmitted infection after vaginal deposition in the cat [10,11], and fresh HIV-infected human peripheral blood leukocytes transmitted infection to SCID mice after vaginal deposition [12,13]. Notably, foreign lymphocytes are able to migrate through vaginal mucosa and reach the iliac lymph nodes of mice [14,15] and HIV-1 infected mononuclear cells are capable of transmigrating through a monolayer of human epithelial cells [16].

Topical microbicides are being developed to reduce the sexual transmission of HIV and other STDs. The presence of both free HIV virions and HIV-infected cells in sexual secretions, and the demonstrated ability of both to transmit infection in diverse animal models, suggests that microbicide candidates should protect against cell-associated HIV as well as cell-free HIV.

Foremost, vaginal microbicides should not injure or disrupt the normal vaginal flora or the vaginal epithelium; thus, microbicides designed to enhance and maintain natural vaginal protective mechanisms merit careful consideration. One natural protective mechanism is the mild acidity found in the healthy vagina (~pH 4) that is generated predominately by the lactic acid produced by vaginal lactobacilli [17,18] and is thought to inhibit harmful flora and some STD pathogens. Two microbicides (BufferGel^®^(ReProtect, Inc., Baltimore, MD) [19-21] and Acidform (TOPCAD, Chicago, IL) [22]), have been developed with the goal of strengthening and maintaining vaginal acidity, by having sufficient buffer capacity to block the alkalinizing action of semen. Another microbicide, CAP, also has acidic buffering properties, which have been postulated to contribute to its activity [23-25]. BufferGel is being tested in an HIV prevention efficacy trial (HPTN 035), and a commercially-available acidifying lubricating gel (Replens^®^) is being used together with a cervical barrier (diaphragm) in an HIV prevention trial [26].

In these studies, we examined the ability of mild acidity to inhibit lymphocyte, monocyte and macrophage motility. We also determined PBMC viability in mildly acidic conditions, and measured the intracellular pH (pH_i_) to determine their ability to defend their cytoplasmic pH. We report the effect of Carbopol^®^, the buffering agent contained in BufferGel, on leukocyte motility and PBMC viability. Finally, we tested the ability of BufferGel to reduce transmission of cell-associated HIV in the HuPBL-SCID mouse model.

## Methods

### PBMC collection

Venous blood and semen were obtained from donors according to procedures approved by the Review Board on the Use of Human Subjects at the Johns Hopkins University. PBMCs were separated on Histopaque^®^-1077 step gradients (Sigma, St Louis, MO). Cells were maintained at 37°C during experiments and were used within 5 hours of collection.

### Monocyte chemotaxis

Gradient purified PBMCs were washed twice in 0.9% saline and resuspended in 0.9% saline at a concentration of 10^8 ^cells/ml. Monocyte chemotactic experiments were performed using microchambers with polycarbonate filters with 5 μm pores (NeuroProbe, Cabin John, MD). The top wells of the microchamber were filled with neutral or acidified RPMI (Gibco, Rockville, MD) supplemented with 1% fetal bovine serum (FBS, Gibco) and then the cell suspension was added. RPMI was acidified with one or combinations of the following acids: acetic acid (JT-Baker, Phillipsburg, NJ), HCl (JT-Baker), or 2-(N-Morpholino)ethanesulfonic acid (MES, Sigma). The bottom wells were filled with RPMI supplemented with 1% FBS at the same pH and a chemoattractant; either 0.1 μM formyl-Met-Leu-Phe (FMLP, Sigma) or 5 μM platelet activating factor (PAF, Sigma). The chamber assembly was then incubated for 90 minutes at 37°C, 5% CO_2_. After incubation, pH in upper and lower chambers was verified to have remained stable (± 0.1 pH units, MI-414-6 pH microelectrode, Microelectrodes Inc., Bedford, NH), the chambers were disassembled, stained with Diff-Quick^® ^(American Scientific Products, McGaw Park, IL) and the number of monocytes that had migrated to the lower side of the filter counted.

### Monocyte, macrophage and lymphocyte chemokinesis

PBMCs separated and washed as above were resuspended in 15 ml RPMI containing either 1% FBS for monocyte or lymphocyte purification or 10% human serum (HS) for macrophage maturation. Monocytes were separated from lymphocytes by reversible fibronectin adherence [27] resulting in ~90% monocyte purity as judged by morphology after Diff-Quick staining. For experiments requiring macrophages, the monocytes were additionally incubated for 3 days in RPMI containing 10% HS [28], and matured macrophages were harvested. For lymphocyte experiments, monocytes were depleted from PBMCs with CD14-coated magnetic beads (Dynal Biotech, Lake Success, NY) according to the manufacturer's protocol. The remaining cells were predominantly lymphocytes (less than 2% monocytes by Diff-Quick staining).

Leukocytes were observed by video microscopy for movement on glass microscope slides (VWR, Bridgeport, NJ) using a published method [29] modified as described below. Monocytes and macrophages were pelleted in a microcentrifuge at 400 G and resuspended in RPMI containing 0.125% Carbopol (BF Goodrich, Cleveland, OH), serum and chemoattractant (1% FBS and 0.1 μM FMLP for monocytes and 10% HS and 0.1 nM FMLP for macrophages). Lymphocytes were resuspended in RPMI containing 1% FBS, 0.1 μM FMLP, and either 50 mM MES or 0.125% Carbopol. The media had previously been adjusted to the desired pH, and the pH of the cell suspensions were verified immediately before placing them under coverslips for video microscopy. Video recordings were made for 15 minutes and then observed at high speed to assess the motility of each of the 50–200 cells contained in a low power field.

### PBMC viability

PBMCs were washed in Hanks' Balanced Salt Solution without Ca^+2 ^and Mg^+2 ^(HBSS; Gibco BRL), resuspended in 10 ml RPMI, and 4 × 10^6 ^cells were placed in microcentrifuge tubes. Tubes were spun at 400 G for 7 minutes and all supernatant removed using drawn out capillary tips, and cells were resuspended in seminal plasma obtained by centrifugation of fresh semen.

PBMCs in seminal plasma (0.05, 0.1, or 0.2 ml) were added to glass vials containing 0.1 g of BufferGel and stirred. Vials were maintained at 37°C during incubations. Additionally, PBMCs were resuspended in seminal plasma with or without 0.2% Carbopol. These samples were observed at pH 7.4 or acidified with HCl to pH 4.5. Each cell suspension was monitored and pH adjusted throughout the timed incubations (1–60 minutes) with the pH microelectrode, and adjusted with 0.1 N NaOH or HCl as necessary to maintain the pH to within 0.03 pH units of the starting pH. After timed incubations, samples were neutralized with 2 ml RPMI with 25 mM HEPES (pH ~8.5) (Gibco BRL) and NaOH (JT-Baker) was used as needed to bring pH to 7.0–7.4. Neutralized cell suspensions were incubated with 2 μM calcein AM and 12 μM ethidium homodimer (Molecular Probes, Eugene, OR) and observed with an epifluorescent Nikon E-800 microscope and live and dead cells counted.

### PBMC intracellular pH

PBMCs were labeled with 50 μM Oregon Green™ 488 carboxylic acid diacetate or 50 μM carboxyfluorescein diacetate (Molecular Probes) for 35 minutes. Fluorescently labeled platelets were removed by four 120 G washes. PBMCs were resuspended at 5 × 10^5 ^cells/ml in either HBSS or a high potassium medium containing 17.8 mM NaCl, 125.2 mM KCl, 8.7 mM Na_2_HPO_4_, 1 mM Ca_2_Cl, 1 mM MgCl_2 _(all JT-Baker), 1.5 mM KH_2_PO_4_, 5 mM D-glucose (both Sigma). Cell viability was determined with trypan blue (Sigma) exclusion. Standard curves were obtained using Nigericin (Molecular Probes), a K^+^/H^+ ^ionophore to equalize the intracellular and extracellular pH of leukocytes in the high potassium medium [30-33]. Aliquots of labeled leukocytes were acidified and fluorescence ratio measurements were taken (ex: 490 nm/440 nm, with em: 520 nm for Carboxyfluorescein and 555 nm for Oregon Green) as a function of time, and ratiometric measurements were made as a function of time on an LS50B fluorometer (Perkin Elmer, Norwalk, CT).

Intracellular pH measurements were also performed using microscopic fluorescent ratiometry using the same dyes and wavelengths as in the cuvette experiments. Aliquots of PBMCs were placed onto Labtek chambered slides (Nalgene-Nunc International, Rochester, NY) coated with 0.1% polylysine (Sigma), labeled and washed. Individual cells were imaged and fluorescence measured in standard and high potassium/ionophore buffer using an Axiovert light microscope (Zeiss, Thornwood, NY) and IP Lab software (Scanalytics, Fairfax, VA).

### HuPBL-SCID mouse model

The HuPBL-SCID mouse was used to model vaginal transmission of HIV-1 as previously described [13,34]. Briefly, SCID mice were administered uninfected human, peripheral blood mononuclear cells (HuPBMC) to the peritoneal cavity one week prior to inoculation, and treated with 2.5 mg Depo-Provera (Upjohn Pharmaceutical, Kalamazoo, MI), which thinned the vaginal epithelium. Vaginal inoculation of 10^6 ^HIV-1-infected HuPBMC followed vaginal administration of 10 μL of PBS, KY jelly, or BufferGel. The inoculated HIV-infected cells, of which between 1 and 5% of the cells are infected with HIV-1 as demonstrated by limiting dilution PCR, were administered on day 10 post-infection. On day 14 following vaginal inoculation, the mice were euthanized and cells from the peritoneal cavity, of both human and murine origin, were collected. HIV-1-infected cells within this population may originate from the infected-cell inoculum, or may be the human target cells from the peritoneal transplant within which the virus has replicated. These cells were placed into culture with PHA-stimulated T cells for co-culture of HIV-1, and were detected by HIV-1 p24 antigen ELISA.

### Statistics

Results from leukocyte chemotaxis and chemokinesis experiments were fit with sigmoidal curves by nonlinear regression. Curves were fit to the four parameter logistics equation [35]:

y = ((d-a)/(1+(x/c)^b^)) + a

where a is the maximum asymptote, b is the slope of the linear region of the curve, c is the midpoint of the linear region, and d is the minimum asymptote. For monocytes and macrophages, the maximum asymptote was set to 100 and for all three cell types, the minimum asymptote was set to 0, since values above 100 and below 0 are not physiological.

Statistical analyses were performed using SPSS^® ^statistical software version 10.0 (SPSS Inc., Chicago, IL). Results from the PBMC viability experiments were analyzed by analysis of variance for the main effects of time and pH and for their interaction. Post hoc pair-wise group comparisons were made using Schéffe's multiple comparison procedure. A linear regression model was developed to determine whether there was a significant difference in viability between PBMCs acidified with HCl and BufferGel, after adjusting for the effects of time and the interaction between time and the acidifying agent. Results for the Hu-PBL SCID mouse model were analyzed with a two sided Fisher's exact test.

## Results

**Figure 1:**
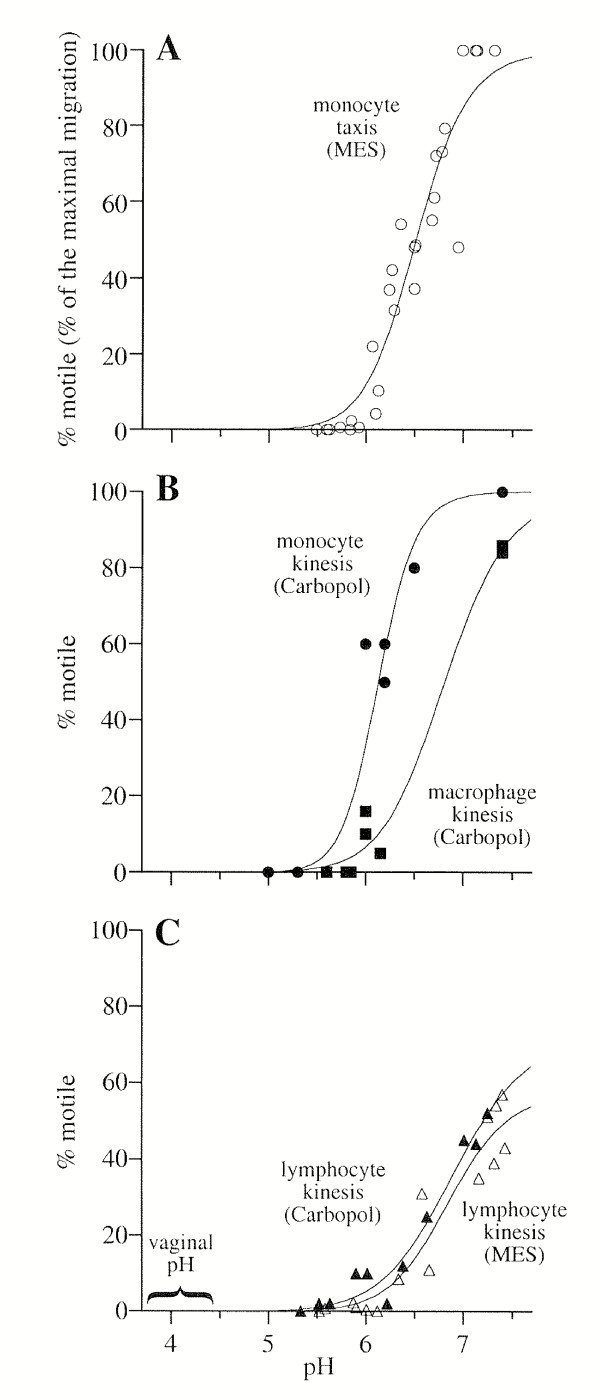
**Leukocyte chemotactic and chemokinetic response as a function of pH. A. **Monocyte chemotactic response, as measured by monocytes migrating through filters of nucleopore chambers, is plotted as a function of pH. Monocyte chemotactic response is normalized to the maximal response for each of four repeats of the experiment (maximum response was between pH 7.0 and 7.3 for each repeat). The cells were in RPMI containing 50 mM MES. Similar results were observed when the media were acidified with buffering systems consisting of 20 mM acetic acid, 20 mM MES and 20 mM acetic acid, and 1 N HCl and when PAF was used as an alternative chemoattractant (data not shown). **B. **Monocyte (circles) and macrophage (squares) chemokinesis, as measured by observing cells migrating on glass slides, is plotted as a function of pH. The percent response was determined by the number of motile cells divided by the total number of cells (50–200) observed at each pH. The cells were in RPMI containing 0.125% Carbopol. **C. **Lymphocyte (triangles) chemokinesis is plotted as a function of pH. The percent response was determined as in B. The cells were in RPMI containing 50 mM MES (open triangles) or RPMI containing 0.125% Carbopol (closed triangles). Nonlinear regression was used to fit a sigmoidal curves for each data set: **A: **R = 0.95; **B: **R = 0.95, monocytes, 0.99 macrophages; **C: **R = .98 lymphocytes with Carbopol, R = .96 with MES. The minimum value was set to 0 for all curves and the maximum value to 100 for monocyte and macrophage curves. The bracket from pH 3.7 to 4.4 on the x axis of panel C indicates the pH range in a healthy, lactobacilli dominated vagina.

Monocyte chemotactic response was observed using chemotactic chambers, over the pH range of pH 5.0 to 7.5, in RPMI acidified and buffered with 50 mM MES. Monocytes attracted by FMLP crawl through a filter separating two chambers filled with media. The percent response normalized to maximal response (pH 7.0–7.5) is plotted for four experiments (Fig. 1A). Chemotaxis was essentially blocked below pH 5.8. Similar results were observed when the media were acidified with buffering systems consisting of 20 mM acetic acid, 20 mM MES and 20 mM acetic acid, and 1 N HCl without additional buffering agent and when PAF was used as the chemoattractant (data not shown).

Since Carbopol consists of micron-sized clusters of lightly cross-linked polymer, it clogged the filters of the microchamber assembly. Therefore, monocyte, macrophage, and lymphocyte chemokinesis were observed in RPMI containing FMLP and 0.125% Carbopol or 50 mM MES by videotaping cells migrating on glass slides. In all cases, nearly all motility ceased below pH 5.8 (Fig. 1B and 1C). The chemokinesis results for monocytes, macrophages, and lymphocytes were similar to the results for monocyte chemotaxis through filters. Although monocytes and macrophages were occasionally able to change shape between pH 5 and 6, progressive motility was never observed below pH 6.0. Lymphocytes exhibited lower maximal motility rates at neutral pH (40–50%) than monocytes or macrophages (85–100%).

**Figure 2:**
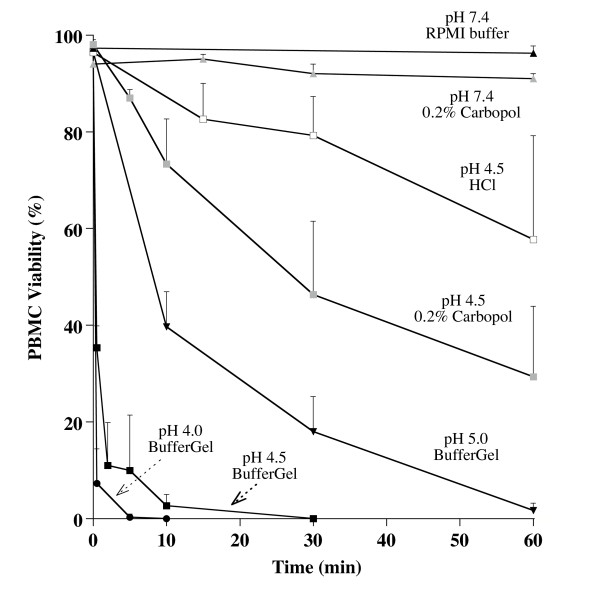
**Viability of PBMCs as a function of time and pH. **PBMCs in seminal plasma were mixed in various volume ratios (1:2, 1:1, 2:1) with BufferGel to give final pH values of 4.0, 4.5 and 5.0. Live PBMCs (as detected calcein-AM) and dead PBMCs (as detected by ethidium homodimer) were counted as a function of time and pH. As a comparison, PBMCs in seminal plasma were mixed with a 0.2% Carbopol (approximately 1/20th the amount in BufferGel), and acidified to pH 4.5 with HCl, or maintained at pH 7.4. Also, PBMCs in seminal plasma without Carbopol were acidified to pH 4.5 with HCl. Each point represents the mean (± SD) of three to six experiments. For experiments with BufferGel, results of analysis of variance for the main effects of pH and time and their interaction were *P *< 0.001. Scheffe's multiple comparison procedure was used to compare pH effects. The pH 5.0 curve is significantly different from the pH 4.0 and 4.5 curves (*P *< 0.001). By linear regression, the pH 4.5 BufferGel curve is significantly different from the pH 4.5 HCl curve (P < 0.001).

To determine if the loss of cell motility was due to killing of the PBMCs, cell viability was tested using a fluorescent live/dead cell assay [36]. As shown in Fig. 2, PBMCs in seminal plasma acidified with HCl to pH 4.5 maintained their viability substantially longer than PBMCs in seminal plasma acidified with BufferGel to pH 4.5 (P = 0.001). When acidified with HCl, 80% of PBMCs were still viable at 30 minutes, but when acidified to this same pH with BufferGel, less than 10% were viable at 5 minutes and none were viable at 30 minutes. To test if this enhanced killing was due to Carbopol, or other constituents or the gel structure of BufferGel, PBMCs were resuspended in seminal plasma containing 0.2% Carbopol (approximately 20-fold lower concentration than in BufferGel) and adjusted to pH 4.5 with HCl, and the viability was measured. This is also relevant for situations where the concentration of Carbopol may be low due to uneven distribution of gel in the vagina. At 30 minutes, 50% of PBMCs in pH 4.5 RPMI containing 0.2% Carbopol were viable. However, PBMCs incubated with 0.2% Carbopol at pH 7.4 had no reduction in viability over one hour.

**Figure 3:**
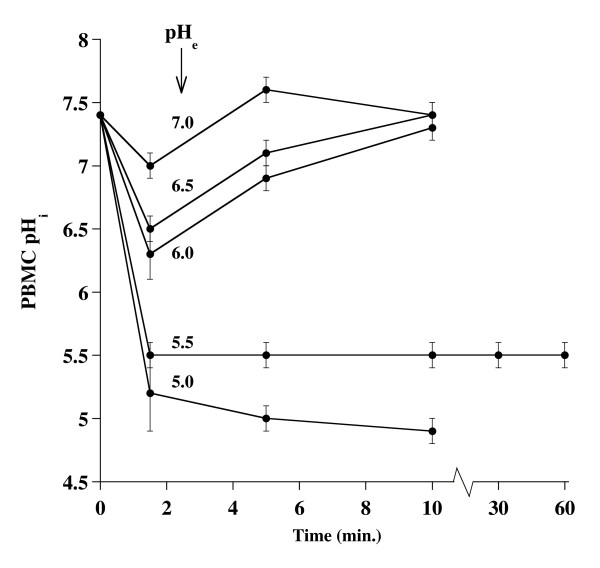
**The effect of extracellular pH on intracellular pH of PBMCs as a function of time. **PBMCs labeled intracellularly with pH sensitive fluorescent indicators (Oregon Green or carboxyfluorescein), were incubated under varying acidic conditions and monitored over time. Upon acidification, PBMC intracellular pH undergoes a rapid decrease to or nearly to extracellular pH. At and above pH_e _6.0, PBMCs recover their normal intracellular pH (7.3) after 5–10 minutes. At pH_e _5.5 and below, PBMCs are unable to recover their intracellular pH. Points are mean (± SD) of three experiments.

PBMC pH_i _was measured as a function of extracellular pH (pH_e_) to investigate potential mechanisms mediating the acid immobilization and killing of PBMCs. At every pH tested, pH_i _equilibrated with pH_e _within 2 minutes (Fig. 3). At pH_e _as low as 6.0, however, PBMCs were able to restore their pH_i _to approximately 7.5 within 10 minutes. However, at pH 5.5 and below, PBMCs were unable to restore their normal pH_i_, even after 60 minutes.

Control experiments using fluorescent microscopy were performed measuring the fluorescence ratio of individual cells and of the surrounding buffer, to detect artifacts due to possible dye leakage out of cells and inadvertent observation of dye in the extracellular buffer. These results showed that dye leakage was not a factor and that, as with the cuvette method, pH_i _dropped to pH_e _at both pH_e _5.5 and 5.0 and pH_i _did not recover (data not shown).

Three groups of mice were challenged with cell-associated HIV in the HuPBL-SCID mouse model. Depo-Provera treated mice were treated with BufferGel, PBS (control), or KY Jelly (gel control) prior to infected-cell challenge. One of 12 mice pretreated with BufferGel became infected with HIV, 12 of 12 mice pretreated with PBS became infected, and 5 of 7 mice pretreated with KY Jelly became infected. BufferGel provided significant protection compared to KY jelly (P < 0.01) and saline (P < 0.0001).

## Discussion

During reproductive years, the pH of a healthy human vagina is usually pH 4–4.5 [37]. However, Masters and Johnson showed that an ejaculate acts as a potent alkaline buffer, which abolishes vaginal acidity within seconds and keeps the vagina neutralized (pH 6–7) for several hours after intercourse [38]. During this time acid-sensitive sperm and microbes can reach their targets or enter the upper reproductive tract. Maintaining the normal acidic condition of the vagina during and after intercourse is thus a potential method for preventing conception as well as STDs.

Here we describe the effect of low pH on the motility and viability of leukocytes that may serve as vectors for sexual transmission of HIV and present data indicating that BufferGel reduces vaginal transmission of cell-associated HIV in the HuPBL-SCID mouse model. We found that monocytes, macrophages and lymphocytes all completely lose progressive motility at a pH slightly below 6.0 (Fig. 1). The results were similar in the presence of a variety of buffering species, including Carbopol, and with a second chemoattractant, platelet activating factor. Our results are consistent with those of Fischer, et al, who showed that lymphocytes lose cytotoxic activity and other immunological functions when the extracellular pH falls to 5.8 [39,40] and with Hill and Anderson who showed that lymphocyte proliferation is abolished at pH < 6 [41].

Acidity dramatically reduced leukocyte viability in this study. When acidified with BufferGel, PBMCs added to seminal plasma were rapidly killed at pH 4.0 and 4.5, and killed more slowly at pH 5.0 (Fig. 2). Both BufferGel and Carbopol alone enhanced acid-killing of PBMCs compared to acidification with HCl alone.

In the SCID mouse model of vaginal HIV transmission, pretreatment of mice with BufferGel, as compared with saline, significantly reduced the transmission of cell-associated HIV. In addition, BufferGel provided significant protection compared to another gel, KY Jelly. We and others have shown similar protection using this model with other microbicide candidates [13,42-44].

Importantly, despite the cell killing potential of acidity especially in the presence of BufferGel, BufferGel has proven to be non-toxic to cervicovaginal epithelium in vivo in two high-dose tolerance Phase I trials [19,45] and in a highly sensitive acute vaginal toxicity mouse model [46]. We hypothesize that this advantageous differential toxicity (toxic to potentially infectious human leukocytes, but non-toxic to human epithelia *in vivo*) may be due to a greater ability of the epithelium to maintain the pH_i _of its surface cells because these cells can export protons to underlying vascularized tissue. In contrast, individual cells or pathogens surrounded by an acidic environment within the vaginal lumen must pump out protons against a high gradient over their entire surface area. Thus, although demonstrably cytotoxic to human white blood cells in vitro, and able to prevent cell-associated HIV transmission in the vagina lumen in an animal model, acidic buffering appears to have minimal or no cytotoxicity to the human genital tract epithelium.

Our results demonstrate that mild acidity inactivates the motility and viability of leukocytes that may act as vectors for sexual transmission of HIV [47,48] and reduces transmission of HIV in a mouse model. These results are consistent with other studies that have shown that the natural vaginal acidity may help reduce HIV transmission in women. Women with abundant vaginal lactobacilli have a lower HIV susceptibility compared to women with bacterial vaginosis [49,50], a condition in which vaginal lactobacilli, and therefore, vaginal acidity, are lost. It has been suggested that reduction of cervicovaginal viral load by physiological vaginal acidity may reduce both male to female, *and *female to male transmission of HIV [51,52]. In a study of HIV infected women, vaginal lavages with a pH < 4.5 showed a trend to contain less cultivatable HIV virus than lavages with a pH ≥ 4.5 (P = 0.08) when tested with the multinuclear-activation galactosidase indicator (MAGI) assay [52] possibly indicating a reduction in HIV load and a reduction in risk of transmission at lower pH values. Moreover, another study showed that exposure to acidic cervico-vaginal secretions reduce HIV viability as detected by cocultivation with PBMCs [51].

To gain understanding about how acid pH mediates the immobilization and killing of leukocytes we measured the pH_i _of PBMCs in acidified medium. Intracellular fluorescent pH probes have been used to measure the pH_i _of human leukocytes in a number of studies [39,53-57]. Most previous studies have concentrated on leukocyte activation and small changes in pH_i _(△pH ~0.5) and minor reductions in pH_e_. One study, exploring leukocyte function near tumors (known to have a low pH_e_), showed that PBMCs incubated for four hours at low pH_e _were unable to maintain their pH_i _below pH_e _6.5 [39]. In our experiments with PBMCs, a significant and continued perturbation of pH_i _was observed at and below a pH_e _of 5.5, indicating that PBMCs are unable to defend their pH_i _below this pH_e _(Fig. 3). At pH_e _of 6.0 and above, PBMCs restored their normal pH_i _within 10 minutes. However, even after 60 minutes, PBMCs were unable to restore their normal pH_i _after exposure to a pH_e _of 5.5. Considering that pH_i _exerts profound influence on the apoptosis pathway [58], intracellular enzyme functions, protein stability, and other molecular interactions, we believe the observed prolonged perturbation of pH_i _is the likely cause of the observed loss of motility and viability at low pH_e_.

## Conclusion

We found that human leukocytes lose motility and viability at pH levels typically found in a healthy vagina and that BufferGel reduces transmission of HIV in HuPBL-SCID mice. These results support the hypothesis that physiologic vaginal flora and vaginal acidity may reduce female to male HIV transmission via HIV-infected cell vectors. Our results further suggest that by helping to maintain acidity that would otherwise be abolished by semen [38,51], BufferGel, a broad spectrum microbicide/spermicide [20,21], may enhance the natural protection of vaginal acidity by killing or immobilizing infected cell vectors in semen, and may thus reduce male to female transmission.

## Competing interests

Cone and Moench are developers of BufferGel and Khanna is an employee of ReProtect Inc. All three hold equity in ReProtect, Inc.

## Authors' contributions

SO participated in the design of the study, conducted pH_i _experiments, assisted with statistical analysis, assisted in data analysis and drafted the manuscript. RC conceived of the study, assisted in data analysis, and helped draft the manuscript. KK designed and conducted the HuPBL-SCID mouse experiments, assisted in data analysis, and helped draft the manuscript. EN conducted viability and chemokinesis experiments, and assisted in data analysis. SW participated in the design of the study, conducted chemotaxis and chemokinesis experiments, and participated in analysis of the data. OJ conducted pH_i _experiments and assisted in data analysis. RM helped design the HuPBL-SCID mouse experiments and assisted in data analysis. TM conceived of and designed the study, supervised experiments, assisted in data analysis, and helped draft the manuscript. All authors read and approved the final manuscript.

## Pre-publication history

The pre-publication history for this paper can be accessed here:


